# Gene Therapy: Will the Promise of Optimizing Lung Allografts Become Reality?

**DOI:** 10.3389/fimmu.2022.931524

**Published:** 2022-07-01

**Authors:** Qimeng Gao, Isabel F. DeLaura, Imran J. Anwar, Samuel J. Kesseli, Riley Kahan, Nader Abraham, Aravind Asokan, Andrew S. Barbas, Matthew G. Hartwig

**Affiliations:** ^1^ Department of Surgery, Duke University Medical Center, Durham, NC, United States; ^2^ Department of Molecular Genetics & Microbiology, Duke University School of Medicine, Durham, NC, United States; ^3^ Department of Biomedical Engineering, Duke University, Durham, NC, United States; ^4^ Division of Cardiovascular and Thoracic Surgery, Duke University Medical Center, Durham, NC, United States

**Keywords:** lung transplant, gene therapy, viral vector, Adeno-associated viral vector (AAV vector), Adenoviral (Ad) vector

## Abstract

Lung transplantation is the definitive therapy for patients living with end-stage lung disease. Despite significant progress made in the field, graft survival remains the lowest of all solid organ transplants. Additionally, the lung has among the lowest of organ utilization rates—among eligible donors, only 22% of lungs from multi-organ donors were transplanted in 2019. Novel strategies are needed to rehabilitate marginal organs and improve graft survival. Gene therapy is one promising strategy in optimizing donor allografts. Over-expression or inhibition of specific genes can be achieved to target various pathways of graft injury, including ischemic-reperfusion injuries, humoral or cellular rejection, and chronic lung allograft dysfunction. Experiments in animal models have historically utilized adenovirus-based vectors and the majority of literature in lung transplantation has focused on overexpression of IL-10. Although several strategies were shown to prevent rejection and prolong graft survival in preclinical models, none have led to clinical translation. The past decade has seen a renaissance in the field of gene therapy and two AAV-based *in vivo* gene therapies are now FDA-approved for clinical use. Concurrently, normothermic ex vivo machine perfusion technology has emerged as an alternative to traditional static cold storage. This preservation method keeps organs physiologically active during storage and thus potentially offers a platform for gene therapy. This review will explore the advantages and disadvantages of various gene therapy modalities, review various candidate genes implicated in various stages of allograft injury and summarize the recent efforts in optimizing donor lungs using gene therapy.

## Introduction

Lung transplantation offers the only hope of curative therapy for patients living with end stage lung disease. Since the initial series of successful lung transplants performed in the 80s, both transplant outcomes and volume have improved substantially. The number of annual lung transplants performed has increased from 69 in 1988 to 4567 in 2016 worldwide (1). In the US, one-year survival following lung transplant is now close to 90%, and one in three recipients survive to ten years following transplant (2).

Despite these favorable trends, many challenges remain. In 2019, the donation rate for lung was 20.8 eligible donors per 100 eligible deaths (3), much lower than those of other solid organs. Sixty-four percent of donor lungs are not recovered due to a variety of reasons, many stemming from the inherent exposure and susceptibility to insults, such as infection, aspiration, trauma, smoking, and ventilator-associated injury. An additional 6.4% of donor lungs were recovered but not transplanted (2). Furthermore, survival after lung transplant remains the lowest of all solid organ transplants. Three major immune-mediated mechanisms account for most lung allograft failures: 1. Ischemic-reperfusion injury (IRI), with subsequent activation of the innate immune system, which is associated with primary graft dysfunction (PGD); 2. Cell-mediated, and to a lesser extent, antibody-mediated adaptive immune activation, which can cause acute rejection; and 3. Chronic graft dysfunction, a process that is poorly understood and thought to be related to inflammation and chronic airway remodeling.

Novel therapies that may maximize donor lung utilization and reduce immune-mediated graft failures are needed; gene therapy has emerged as a potential strategy for organ rehabilitation and optimization. The past decade has seen significant progress in gene therapy development as several products are now approved for clinical use (4). Concurrently, novel organ preservation strategies in normothermic *Ex Vivo* Lung Perfusion (EVLP) are gaining popularity after promising results in clinical trials (5, 6). EVLP techniques maintain lung allografts in a physiologically active and ventilated state, and thus offer an optimal platform for delivery of interventions such as gene therapy. In this review, we present progress in the use of gene therapy in lung transplantation, and highlight some important considerations related to the routes of delivery, vector choice and potential target genes.

## Gene Therapy Approaches

The core of gene therapy is the use of exogenous DNA in promoting, modifying, or silencing protein expression. Gene therapy initially gained traction due to its potential to offer a durable cure for many inherited monogenic disorders with a single treatment (7). Although the field’s progress has ebbed and flowed, the past decade has witnessed a renaissance in gene therapy with several products receiving FDA approval following generations of efforts, including two AAV-based *in vivo* therapies: Luxturna^®^ for Leber’s Congenital Amaurosis and Zolgensma^®^ for spinal muscular atrophy (8). There is ongoing investigation to utilizing genetic therapies in other fields of medicine, including lung transplantation.

**Figure 1:**
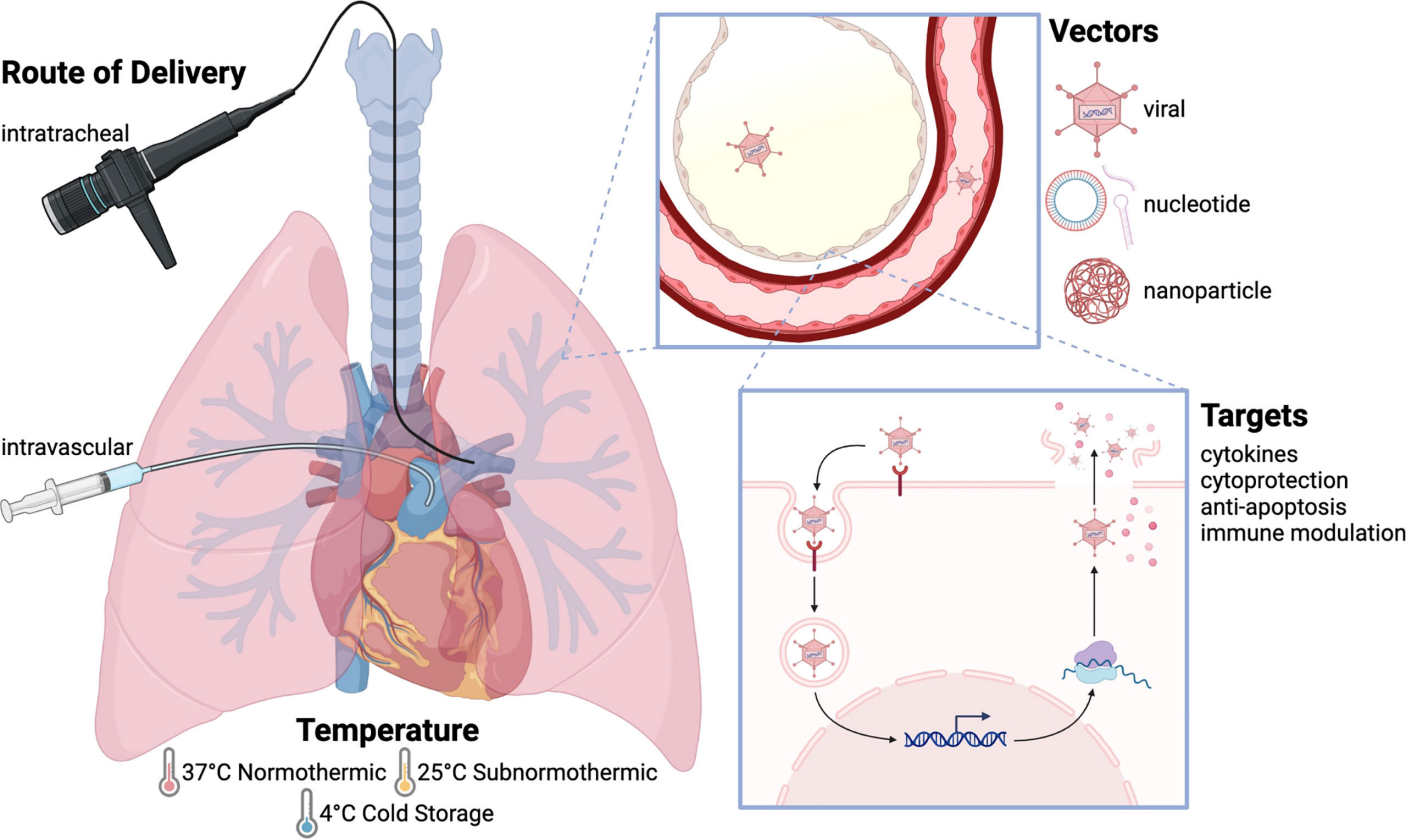
Approaches to gene therapy in lung transplantation. The basic principle of gene therapy is that gene sequences are delivered to target tissues using a vector, which is then taken up by these tissues allowing for gene expression. Gene therapy can be delivered using multiple routes, temperatures, and vectors, with numerous gene targets and uses. Intratracheal delivery is most commonly used, as it allows for localized treatment without systemic effects, although intravascular delivery has been used to target the vascular endothelium. Gene therapy is often delivered at physiologic temperature (37°C), although studies have also been performed at subnormothermic temperature (25°C) and cold storage temperature (4°C). Adenovirus is the most widely employed vector. Other viral vectors include AAV, lentivirus, and sendai virus. Nucleotide-based vectors include naked plasmid, shRNA, and siRNA, and nanoparticles have also been used to deliver gene products. The transfected genes include anti-inflammatory cytokines (IL-10, TGF-β), pro-inflammatory cytokines (IL-1, TNF-α), cytoprotective genes (NOS), anti-apoptotic molecules (caspase-3, FasL), and immune modulatory molecules (CTLA4-Ig, IDO).

Several features unique to the field of transplantation are advantageous for genetic-based therapeutics (**Figure 1**). First, transplant patients are immunosuppressed, and it is hypothesized that innate immunity and subsequent immune activation against gene therapy vectors or foreign protein products can reduce therapeutic efficacy (9). Studies in both clinical and experimental animal models have demonstrated that immunosuppression facilitates gene therapy delivery and enhances gene expression (10–13). Second, considerable research in vector engineering has focused on optimizing tissue tropism, as most *in vivo* gene therapies are delivered systemically (14, 15). In transplantation however, organs are preserved *ex vivo* following procurement, which provides a unique opportunity for isolated organ-targeted treatment; this period of isolation could potentially enable utilization of higher vector doses and reduce the incidence of systemic adverse effects, such as those observed in early gene therapy clinical trials (16).

### Temperature of Delivery

Although administration of gene therapy to donor animals has generally been successful (11), it is not clinically relevant, as the treatment of human organ donors would raise significant ethical concerns. Thus, an ideal window for gene delivery to donor organs is between donor cross-clamp and reperfusion in the recipient. In lung transplantation, most grafts are preserved at 4°C or less, while clinical EVLP protocols to date preserve lung grafts at 37°C. In recent years, others have proposed alternative preservation strategies that may reduce inflammation where lungs were maintained with EVLP at sub-normothermic temperatures between 10 to 25°C (17–19).

Studies have demonstrated that gene expression can be detected in the target organ following reperfusion when gene therapies are delivered at different temperatures, ranging from 4°C to 37°C, across multiple organ systems, and in a variety of models (20–26). Thus, even though gene expression relies on substantial cellular machinery for transcription and translation at physiological temperature, there is evidence suggesting that gene delivery can be achieved during cold storage with subsequent transgene expression. Delivery of genes during static cold storage could offer an opportunity for translation to the clinical setting, as most organs are currently stored on ice.

Conceptually, preservation of the lung allograft closer to physiological temperature may improve vector uptake and gene expression as the graft is metabolically more active. After delivery of adenoviral vector encoding human IL-10 (hIL-10), Cypel et al. found increased hIL-10 concentrations in lung tissue in porcine lungs prior to reperfusion only when maintained on EVLP but not when kept under cold static conditions (23). Cassivi et al. found minimal transgene expression in donor lungs that received gene therapy during cold storage when comparing target protein expression at reperfusion between therapy delivery to the donor animal six hours before procurement, or after procurement during cold storage (27).

Ultimately, the optimal donor organ storage conditions for gene delivery is likely to be determined by the target gene of interest. For example, if the target is a mediator of IRI and a high concentration of the desired protein product would be needed at the time of reperfusion, then gene therapy during EVLP could be desirable in order to maintain metabolic activity for 6-8 hours prior to implantation. In contrast, gene delivery during cold storage could be sufficiently effective if targeting a later event such as acute or chronic rejection.

### Routes of Delivery

Gene transfer to the lung allograft is generally achieved *via* the airway or intravascularly. Intra-tracheal delivery gives direct access to lung epithelial cells and, since the airway compartment is relatively isolated, the risk of systemic spread and off target effects upon reperfusion is minimized. In fact, most studies on lung-targeting gene therapy for the treatment of cystic fibrosis and α1-antitrypsin deficiency favor this approach (14, 28–30). Unsurprisingly, most studies on gene therapy in lung transplant have also elected to use the airway delivery approach (11, 23, 27, 31–33), because this route of delivery produces significantly greater gene expression in the lung overall, predominantly in respiratory epithelium, compared to other approaches (34, 35). However, one concern related to gene delivery *via* the airway is that it poorly targets the vascular endothelium (36). Vascular endothelium is the interface between the donor organ and the recipient circulation, and thus plays an important role in IRI and other immune mediated injuries.

As a result, investigators have turned to the intravenous delivery route, hoping to achieve better gene transfer in vascular endothelial cells. Thus, therapy is typically delivered *via* the pulmonary artery *ex vivo* or systemically *in vivo*. Early studies have demonstrated the feasibility of intravascular gene transfer to lungs *in vivo* or *ex vivo* using adenoviral vectors in rodent and large animal models (36–39). Target genes are detected in vascular endothelium and sometimes airway epithelium, but not alveolar epithelium, which is more readily transfected during trans-airway delivery (36, 40). In most cases, the efficiency of gene delivery was low, and off target delivery was detected in remote sites even when the viral vectors were injected *ex vivo* (34, 39).

Lastly, the lung is dually perfused by the pulmonary artery and the bronchial arteries. The bronchial artery receives about 1% of the cardiac output and provides systemic blood supply to the trachea and bronchi (41). Therefore, the bronchial artery can theoretically be utilized for gene delivery. There has been some speculation that gene delivery *via* the bronchial artery may improve targeting to the bronchial airways (36, 42), but further research is needed to demonstrate the efficacy of gene delivery through the bronchial artery.

### Choice of Vector

Two general approaches exist to transfer genetic material into a target cell—viral and non-viral. Both have been applied in gene therapy studies in lung transplant (35, 43). The choice of vector is important, especially given the vector-associated safety issues in early gene therapy clinical trials (44). In general terms, viral vectors transduce the genes efficiently but can pose some safety concerns, while non-viral vectors tend to be safe and less immunogenic but are less efficient at gene transfer. Recent years have seen a shift in the field of gene therapy in terms of the choice of vector: the field has turned away from adenoviral-based vectors towards Adeno-associated virus (AAV) – based vectors for *in vivo* gene delivery. Lentiviral vectors are currently the leading platform for ex vivo gene modification in primary cell lines such as T cells (4). Non-viral vectors have also emerged, as the first siRNA-based drug Onpattro^®^ delivered using lipid nanoparticle (LNP) technology was approved for the treatment of hereditary ATTR amyloidosis (45).

#### Adenoviral Vector

Adenovirus (Ad) is one of the first extensively studied vectors and the first effective *in vivo* gene therapy tested clinically (46). Early experiments in gene therapy for lung transplant and other solid organs have relied heavily on adenovirus-based vectors (20, 23, 32, 33, 35, 47). Ad vector has several advantages: 1. its ability to infect both non-dividing and dividing cells with high transduction efficacy; 2. the availability of scalable production systems; 3. a large insertional capacity for the target gene, around 7-8 kb; and 4. broad tissue tropism.

One major issue associated with Ad vector is its inherent high immunogenicity—unrelated to the transgene expression. A strong innate immune response against the capsid protein is well documented in animal models (48) and may trigger significant cytokine storm in susceptible individuals (44). Subsequently, robust host adaptive immune responses to the transgene product reduce the longevity of gene transfer. In a rat liver transplant model, Olthoff et al. successfully delivered CTLA4Ig to the donor liver using an Ad vector, although minimal levels of CTLA4Ig were observed beyond 20 days post-transplant (20). In a porcine lung transplant model, Machuca et al. delivered Ad vector encoding hIL-10 during EVLP. Although they were able to detect hIL-10 in the perfusate while on EVLP as early as 12 hours following Ad vector administration, serum levels of IL-10 post-transplant decrease drastically within the first week (33). In humans, the effective expression of Ad vector *in vivo* peaks at 1 week and is generally limited to about 2 weeks (49). Related to Ad’s immunogenic nature, the efficacy of repeat administration of Ad vectors is limited by the development of neutralizing antibodies directed against the vector following initial delivery. Additionally, pre-existing immunity against various Adenovirus serotypes exists population-wide, thus limiting the wide adoption of Ad vector (50).

As a result, Ad vector is increasingly being utilized for the treatment of cancer or vaccines, where a strong immune response is desired. In transplant, adenovirus vectors may remain of interest if rapid onset and short-term gene expression is desired (49).

#### Adeno-Associated Viral Vector

AAV is a nonpathogenic virus that requires co-infection with a helper virus to replicate in host cells. Although it can infect both dividing and growth-arrested cells, AAV infection causes no known disease in humans. Recombinant AAVs contain no viral proteins and cause less inflammatory response compared to Ad vector (15, 51, 52). Perhaps because of its excellent safety profile (53), AAV is currently the leading platform for gene delivery for the treatment of a variety of human diseases. Hundreds of phase I/II/III trials have been conducted to date using recombinant AAV vectors, with several AAV vector-based gene therapy products approved in Europe and USA (15, 54).

Additionally, AAV vectors are highly versatile with wide-ranging tissue tropism among serotypes. AAV vector platforms have been developed to target the central nervous system (CNS), eyes, liver, lung, heart, and muscle in a variety of preclinical and clinical models (50). Following delivery, AAV vectors typically persist in host cells in a stable episomal form and rarely can integrate at the site of double-strand breaks. (55, 56).

Because AAV is a single-stranded DNA (ss-DNA) virus, the ssDNA encoding the target gene needs to be converted into a double-stranded DNA (dsDNA) before transcription. As a result, transgene expression only rises to detectable levels in 1-2 weeks. In comparison, gene expression following Ad vector delivery becomes detectable within several hours. One alternative is the use of a self-complementary design, in which the packaged DNA product within the AAV vector undergoes intramolecular base pairing to form dsDNA (57). This way, transcription can begin as soon as the DNA reaches the nucleus. One disadvantage of the self-complementary design is that it further limits the packaging capacity of AAV to 3.3 kb (58).

In contrast to Ad vector, AAV vectors have been shown to achieve long-term transgene expression in experimental models and clinically. In a canine model of Hemophilia A, animals treated with AAV gene therapy have sustained factor VIII activity 10 years from the initial treatment. Clinical trials of AAV gene therapy in the liver have also demonstrated long-term efficacy (59, 60).

Of note, there are several important disadvantages associated with AAV vectors. Firstly, the target gene delivered by AAV is generally limited to less than 5 kb. However, the use of dual overlapping vector strategies could circumvent this limit (61). Secondly, transgene expression following AAV delivery generally does not reach clinically relevant levels until 1-2 weeks. Even with the self-complementary design, expression remains minimal within the first 24-48 hours. Such a timeline makes AAV a less-than-ideal vector candidate if IRI is the primary target of intervention.

To date, few AAV gene therapy studies have been published in lung transplant models. A recent study described the delivery of reporter gene using AAV8 during hypothermic oxygenated machine perfusion (HOPE) in a liver transplant model (62), and our group has similarly reported AAV9 delivery of a reporter gene to donor liver and lung during static cold storage (25, 26). Overall AAV has demonstrated its versatility and safety profiles in clinical trials and is thus most readily translatable for clinical uses. However, AAV’s utilization in targeting IRI specifically is likely limited due to its delayed action.

#### Lentiviral Vector

Lentiviral vectors have been increasingly used for gene therapy due to a number of unique characteristics: 1. They transduce non-dividing cells and offer efficient and durable gene expression (63, 64). This feature is appealing for lung applications given than 95% of all lung cells are quiescent (65). 2. Lentiviral vectors have a large packaging capacity (up to 9 kb). 3. They trigger minimal inflammation and immunogenic responses—a major advantage compared to adenoviral vectors (63). Their low immunogenicity profile allows for repeated doses with minimal decrease in efficacy (66). 4. Lentiviral vectors can be pseudotyped by altering their surface receptor recognition elements to target various cell types (67).

Lentiviral vectors have been used successfully to transfect lungs in murine models (65, 68, 69). In mice, airway delivery of lentivirus-encoding luciferase led to robust luciferase activity that was stable over 15 months, highlighting the potential of stable long-term transgene expression using lentiviral vectors in lungs. Of note, gene transduction occurred mainly in alveolar macrophages (70). Furthermore, Cooney et al. reported CFTR delivery *via* aerosolized FIV-based lentiviral gene therapy in a porcine cystic fibrosis model (71) with evidence of functional CFTR 2 weeks following gene therapy, demonstrating the efficacy of lentiviral vectors in a large-animal model.

Lentiviral-based gene therapy has also been tested in lung transplantation models. The Toronto group demonstrated in a mouse model that lentivirus-mediated trans-airway gene transfer to the donor lung results in persistent transgene expression up to 6 months in a syngeneic model (72). Of note, the efficacy of gene delivery to the donor lung was comparable whether the vector was given to the donor pretransplant or to the recipient posttransplant. A later study by the same group demonstrated the efficacy of a combination of *ex vivo* and *in vivo* lentiviral vector administration to the recipient lung (73).

Despite these advances, several concerns limit the application of lentiviral vectors to gene therapy. First, there is a risk of generating a replication-competent virus. Several modifications have been made to lentiviral vectors, including splitting the viral genome into separate plasmids as well as removing accessory virulence factors (64, 74). Second, there is a potential to cause insertional mutagenesis at the site of vector integration, leading to malignant transformation in the treated cells (75, 76). Lastly, large scale production of lentivirus for clinical use can be challenging (77). As such, optimization in pre-clinical models remains essential prior to utilizing lentiviral vectors in transplantation and machine perfusion.

#### Non-Viral

Given the intrinsic limitations (e.g. amount of genetic material deliverable, differential cell transfection) and safety considerations (e.g. infectious, oncologic) of viral vectors, non-viral based gene therapies have been explored. Naked delivery, the delivery of DNA without delivery agents, has shown overall mixed results with both plasmid-based therapy (78–80) and siRNA-based gene therapy (81, 82). The major limitations of these strategies remain poor stability and rapid degradation (45, 83), limited uptake of various types of cells in the lung (84), and possible off-target effects of unformulated siRNA (85).

Several chemical carriers have thus been developed to overcome those limitations including lipid-, peptide-, polymer-, and nanoparticle-based carriers (8, 86–88). Of those approaches, nanoparticle-based gene therapy is most promising with currently more than ten FDA-approved lipid nanoparticle (LNP) drug delivery systems (45). LNPs readily achieve delivery of large amounts of genetic information, are easy to scale up, and cause minimal inflammation and immune responses (89, 90). Importantly, several groups recently reported generation of lung-targeting LNPs (91–93), a major step in developing lung-specific gene therapies.

## *Ex vivo* Lung Perfusion Platform

The past decade has witnessed a strong push for the expansion of the donor pool. This is driven primarily by increased utilization of organs that have been thought to confer an increased risk of graft failure. Transplant centers are increasingly using donation after cardiac death (DCD) donor lungs and adopting a more liberal approach to donor lung acceptance, including using long cold ischemic time organs and organs from donors over 60-years-old, Hepatitis C Virus (HCV) positive donors, and donors with a significant smoking history (94–97).

Coincidentally, the adoption of novel preservation technology, i.e. *ex vivo* lung perfusion (EVLP), has allowed improved evaluation of donor lungs under physiological conditions, which is particularly useful for traditionally marginal organs. In fact, a recent multicenter trial in the US described a portable EVLP device that has led to an 87% donor lung utilization rate, four times the rate observed when lungs are preserved on cold ice (98). In addition to its capacity to evaluate lung allografts, the EVLP platform also provides a window to administer therapies that recondition donor lungs and minimize injury following reperfusion. The Toronto group has previously examined the efficacy of high dose antimicrobials on bacterial and fungal infections (99), ultraviolet C light on controlling hepatitis C viral concentration (100, 101), and Rituximab on depletion of B cells latently infected with Epstein Barr virus (102) in discarded human lungs. Others have investigated various anti-inflammatory compounds in animal models (103–105).

EVLP provides an optimal platform for the delivery of gene therapy. Airway and vasculature are both accessible while on EVLP and thus available for vector administration. As the organs are maintained under physiologically active conditions, vector uptake and transgene expression can readily occur. Additionally, any downstream effects or potential side effects associated with gene therapy can be monitored. Aside from the Ad vector, the initiation of transgene expression following gene therapy administration can take days. While most clinical EVLP protocols limit organ preservation to 12 hours, it may be possible to prolong this window of intervention using EVLP with additional external nutrition and dialysis support to the donor graft (106, 107), thus allowing a longer window for transgene expression.

Several aspects of EVLP merit special consideration regarding the delivery of gene therapy. First, various EVLP devices and protocols exist, but all maintain the organ at physiological temperature. As a result, most studies on gene therapy during EVLP are done under normothermic conditions. However, EVLP performed at subnormothermic temperatures may be useful in delivering gene therapy. Our group recently reported less inflammation to the donor graft when EVLP was performed at 25 °C compared to 37°C in a rat model (19). A similar result was also noted by Arni et al. (108). Gene delivery during subnormothermic EVLP has not yet been attempted, and more research is needed to determine the optimal temperature for delivery and subsequent transgene expression. Secondly, several EVLP protocols use RBC-based perfusate. In the case of Ad vector, various blood components, including RBC, PBMC, plasma and serum, have been found to interfere with vector transduction of cell lines *in vitro* (47). Therefore, EVLP protocols with RBC-free perfusate may be ideal to minimize interference of gene delivery. Third, if the vector is to be delivered through the vasculature, the circulating volume of perfusate is likely to play a significant role in the vector dose (and therefore cost) required to induce effective transduction. Lastly, while there has been recent enthusiasm regarding the utility of EVLP, not all donor organs require EVLP prior to implantation. EVLP preservation requires additional time and resources compared to static cold storage. Gene delivery to donor organs does not necessarily require EVLP, though EVLP could help facilitate transgene expression when targeting processes occurring soon after reperfusion.

## Current Status of Gene Therapy in Lung Transplant

**Figure 2:**
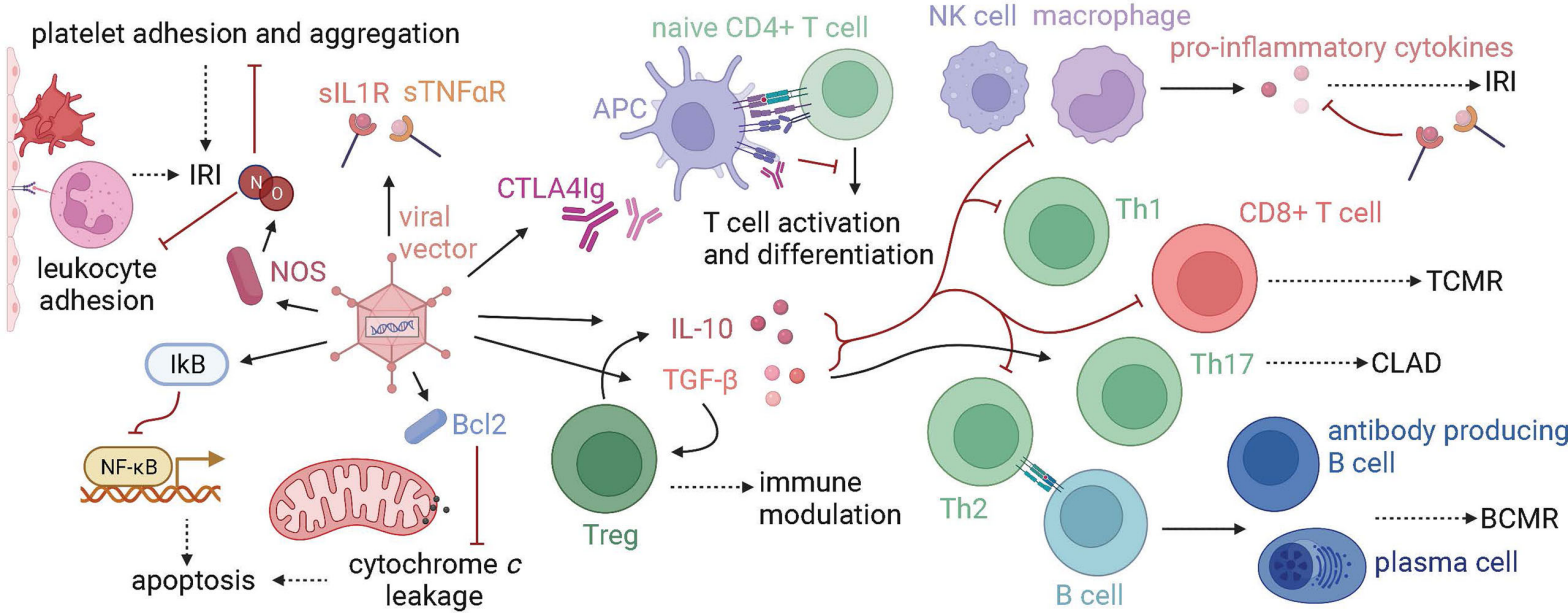
Gene therapy targets in lung transplantation. Graft injury at many timepoints in transplantation, from procurement to years following transplant, has been targeted using gene therapy. Proteins that modulate the immune system, prevent apoptosis, or protect the endothelium have been transfected using vectors, namely viral vectors such as adenovirus. Anti-inflammatory cytokines such as IL-10 and TGF-beta decrease innate immune responses, T cell effector and helper functions, and B cell costimulation, which makes them a target in the treatment of IRI, TCMR, BCMR, and CLAD. Tregs are activated by TGF-beta, produce IL-10, and have immune-modulating functions. Other immune-modulating proteins such as CTLA4Ig, which blocks T cell activation, and soluble IL1 and TNF-alpha receptors (sIL1R, sTNF-alphaR), which block the downstream effects of proinflammatory cytokines, have been used in gene therapy models. NOS is a target for ameliorating IRI, as the production of NO decreases both platelet and leukocyte adhesion. Anti-apoptotic molecules, such as IkB and Bcl2 have also been used in IRI models. (IRI, ischemia-reperfusion injury; TCMR, T cell-mediated rejection; CLAD, chronic lung allograft dysfunction; BCMR, B cell-mediated rejection).

Gene therapy has been utilized to target several challenges in lung transplantation, including IRI, acute rejection, and chronic graft dysfunction (**Figure 2**, **Table 1**).

**Table 1:** Target genes used in previous gene therapy studies in lung transplant.

Mechanism of Injury	Pathway	Vector	Route of Delivery	Citation
IRI	IL-10	Adenovirus	IV *in vivo*	(110)
	IL-10	Adenovirus	IT *in vivo*	(111)
	IL-10	Adenovirus	IT *in vivo*	(112)
	IL-10	Adenovirus	IM *in vivo*	(34)
	IL-10	Adenovirus	IT *in vivo* and *ex vivo*	(32)
	IL-10	Adenovirus	IT *ex vivo*	(23)
	IL-10	Adenovirus	IT *ex vivo*	(33)
	TGFβ	Naked plasmid	IM *in vivo*	(80)
	TGFβ + IL-10	Naked plasmid	IM *in vivo*	(79)
	sTNFαR	Adenovirus	IT *in vivo*	(118)
	sIL1R	Adenovirus	IT *in vivo*	(119)
	NOS	Adenovirus	IT *in vivo*	(120)
	HSP70	Adenovirus	IT *in vivo*	(121)
	IκB	Adenovirus	IT *in vivo*	(123)
	Bcl2	Adenovirus	IT *in vivo*	(122)
	Caspase-3	shRNA	IT *in vivo*	(124)
	P38α	shRNA	IT *in vivo*	(125)
	Fas	siRNA	IT *in vivo*	(126)
Acute rejection	IL-10	Plasmid	IT *ex vivo*	(127)
	IL-10	Lentivirus	IT *in vivo*	(72)
	vIL-10	Naked plasmid	IT *in vivo*	(129)
	vIL-10	Adenovirus	IT *in vivo*	(130)
	vIL-10	Liposome	PV *ex vivo*	(128)
	IL-10	Adenovirus	IT *ex vivo*	(33)
	TGFβ	Naked plasmid	IT *in vivo*	(131)
	TGFβ	Lipid	IV *ex vivo*	(132)
	TGFβ	Adenovirus	IM	(133)
	TNFα inhibitor	Adenovirus	IM	(134)
	TGFβ + IL-10	Adenovirus	IM	(135)
	NOS	Adenovirus	IT *in vivo*	(136)
	ODN	Sendai virus-liposome complex	IT *in vivo*	(137)
	FasL	Lipid	PV *in vivo*	(138)
	IDO	Adenovirus	IT *in vivo*	(141)
	IDO	Lipid	IT *in vivo*	(142)
	CTLA4Ig	Adenovirus	IT *in vivo*	(143)
	Class I and II MHC	Lentivirus	IV *ex vivo*	(144)
CLAD	IL-10	Adenovirus	IV pump, *in vivo* (recipient)	(145)
	IL-10	Lentivirus	IT *in vivo*	(70)
	IL-10	Sendai virus	Direct injection into the graft *in vivo*	(146)
Acute and chronic rejection	IL-10	Lentivirus	IT *ex vivo* and *in vivo* (recipient)	(73)
	HIF1α	Adenovirus	IT *in vivo*	(147)

### Ischemia-Reperfusion Injury

IL-10 is the most widely employed target of gene therapy approaches to mitigate IRI given its broad anti-inflammatory properties. Exogenous administration of IL-10 has previously demonstrated the ability to attenuate lung IRI (109). In murine transplant models, administration of IL-10 gene therapy is most often delivered intratracheally to the donor *in vivo* using an Ad vector, which results in improved post-reperfusion lung function, with decreased expression of TNF-α, IFN-γ, and nitric oxide synthetase (NOS) (110–112). In addition, the delivery of IL-10 gene therapy to the donor graft *in vivo* causes a shift from necrosis to apoptosis as the dominant mode of IRI-induced cell death in a rat lung transplant model (113). Intramuscular Ad vector encoding IL-10 similarly ameliorates IRI in small animal lung transplantation models but was found to require a higher vector titer and trigger systemic effects that local delivery avoids (114).

As detailed in earlier sections, one challenge of the use of Ad vector is a vector-induced inflammatory response, especially when administered *in vivo* to the donor, in both small and large animal models (11, 115). In fact, even empty Ad virion post gene delivery can continue to serve as a significant source of stress and inflammatory response (116). One potential solution is to allow sufficient time for IL-10 transgene expression in the donor prior to organ retrieval so that by the time of retrieval, the anti-inflammatory effect of IL-10 has reversed the vector-induced inflammation. de Perrot et al. also demonstrated methylprednisolone given prior to Ad vector administration reduces these inflammatory effects (11).

An alternative approach is to remove the impact of donor immune system and deliver Ad vector *ex vivo*. A comparison of *in vivo* and *ex vivo* intratracheal delivery of AdhIL-10 in a porcine transplant model saw improved lung function and decreased inflammation on histology in the *ex vivo* group despite cytokine levels comparable to those of the *in vivo* group, perhaps due to the absence of circulating leukocytes in *ex vivo* perfusion circuits (32). The same group also demonstrated the utility of *ex vivo* intratracheal delivery in discarded human lungs, reproducing the findings observed in small animal models (23). AdhIL-10 delivery to discarded human lungs resulted in maintenance of pulmonary physiologic parameters, decreased IL-1β and IL-8, and restoration of tight junctions on histology, indicating successful suppression of inflammatory injury in lung allografts (23).

TGF-β is generally considered an anti-inflammatory cytokine, although its roles in fibrosis and promoting Th17 cells are also well established (117). As applied to IRI, TGF-β gene transfer has been utilized as monotherapy and in combination with IL-10 transduction. IM administration of naked plasmid encoding activated TGF-β1 prior to lung transplantation and following prolonged ischemia decreases cytokines such as TNF-α, IFN-γ, and IL-2, reduces neutrophil sequestration, and improves IRI in a small animal model (80). Unsurprisingly, the combination of TGF-β1 and IL-10 transfection potentiates amelioration of IRI and reduction of pro-inflammatory cytokines (79). Conversely, other studies have utilized Ad vectors that produce inhibitors of pro-inflammatory cytokines such as TNF-α and IL-1. Soluble TNF-α and IL-1 receptors inhibit the inflammatory effects of these cytokines, thus ameliorating IRI in rodent models (118, 119).

Other than targeting IL-10 and TGF-β, both of which are broadly anti-inflammatory cytokines, researchers have investigated specific pathways implicated in IRI or cell death. Ad vectors have been used to deliver several cytoprotective genes in a number of IRI small animal studies. During IRI, nitric oxide synthase (NOS) in the endothelium produces less nitric oxide (NO), which, under normal conditions, inhibits leukocyte adhesion and platelet adhesion and aggregation. Systemic *in vivo* administration of endothelial NOS using an Ad vector improved oxygenation and reduced neutrophil sequestration 24 hours posttransplant (120). Similar results were seen with the delivery of heat shock protein 70 (HSP70), a protein known to increase resistance to IRI, to the donor lung (121). Anti-apoptotic proteins have also been targeted in lung IRI experiments. IκB, an inhibitor of NFκB, and Bcl-2, a protein that prevents cytochrome *c* leakage from the mitochondria, both decrease induction of apoptosis and ameliorate IRI when delivered using an Ad vector in rodent models (122, 123). Short hairpin RNA (shRNA) and small interfering RNA (siRNA) are other delivery mode that has been used to suppress apoptosis. Use of intratracheal shRNAs targeting caspase-3 and p38α, a mitogen-activated protein kinase, prevents apoptosis and protects the lung from IRI (124, 125). Similarly, intratracheal Fas siRNA decreases pulmonary cell apoptosis and edema without decreasing cytokine release or neutrophil infiltration (126).

### Acute Rejection

The role of several anti-inflammatory cytokines has been evaluated in acute rejection (AR) models. In a major MHC mismatched rat lung transplant model, Oishi et al. showed decreased AR scores, pulmonary edema, hemorrhage, and necrosis along with decreased inflammatory cytokines 6 days posttransplant in those that received hIL-10 plasmid *ex vivo* (127). Similar results were demonstrated by Hirayama et al. in a minor MHC mismatch model, where hIL-10 was delivered using a lentiviral vector given both pretransplant to donors and posttransplant to recipients (72). In another set of experiments, Itano et al. initially did not see any difference in physiologic parameters or rejection score when viral IL-10 (vIL-10) was delivered endobronchially using a naked plasmid to the donor lung (128); however, vIL-10 delivery *via* the pulmonary vein using a liposomal vector did result in improvement of physiologic parameters and AR score (129). Interestingly, transgene expression was confirmed in both cases, but semi-quantitative western blot showed better expression of vIL-10 when delivered *via* a liposomal vector,. In a later study, Osada et al. delivered vIL-10 using an Ad vector *in vivo* to the airway and failed to see any significant difference in AR score 6-day posttransplant, despite successful transgene expression (130). The inherent immunogenicity of the Ad vector may add to the alloimmune-mediated damage and interfere with vIL-10’s effect on the acute rejection phenotype. Most recently, Machuca et al. examined the delivery of AdhIL-10 in a porcine lung transplant survival model. At day 7 posttransplant, lungs that received IL-10 gene therapy had better gas exchange and lower inflammation scores. Additionally, recipient lymphocytes from the AdhIL-10 group show less proliferation and IFN-γ production on mixed lymphocyte reaction (33). Apart from IL-10, the delivery of several other anti-inflammatory cytokines using gene therapy has been evaluated in acute rejection models with mixed results. Endobronchial plasmid delivery of TGF-β1 resulted in improved PaO_2_ and decreased rejection scores in mice (131). *Ex vivo* delivery of lipid-mediated TGF-β1 improved oxygenation without improving rejection scores (132). Furthermore, several studies delivered genes encoding anti-inflammatory cytokines intramuscularly, and evaluated the impact of systemic production of those cytokines on lung transplant outcomes (133–135).

As in IRI experiments, cytoprotective genes have also been utilized in AR studies with variable success. Transbronchial administration of adenoviral eNOS does not reduce AR scores despite increase in NO production (136). In contrast, Sendai virus-liposome complex-mediated transfection of oligo-deoxynucleotide (ODN), an NFκB inhibitor, improved graft function and oxygenation and decreased rejection scores (137). Targeting anti-apoptotic molecules such as FasL improved graft function and rejection scores when paired with cyclosporine A (138).

Another approach is to deliver transgenes that specifically target adaptive immunity. IDO degrades tryptophan and suppresses T cell responses *via* tryptophan metabolites (139, 140). Treatment of donor lungs with viral or non-viral vectors encoding IDO inhibited T-cell response and decreased AR scores, cell necrosis, and apoptosis (141, 142). CTLA4Ig inhibits T cell costimulation by disrupting B7-CD28 interaction, thus suppressing T cell activation. CTLA4Ig delivery to donor lung allografts using an Ad vector, did not reduce lymphocyte infiltration or AR scores on day 4 posttransplant in spite of transgene expression (143). Lastly, a German group expanded on this approach by attempting to silence graft major histocompatibility complex (MHC) using a gene therapy approach in a porcine model. MHC mismatch can trigger alloimmune-mediated responses and thus deletion of MHC on graft endothelium could reduce graft immunogenicity. The authors delivered a lentiviral vector encoding short hairpin RNA (shRNA) targeting beta-2-microglobulin and class II-transactivator transcripts during EVLP. Using this approach, they were able to demonstrate the deletion of swine leukocyte antigen (SLA) from the majority of endothelial cells following vector delivery *in vitro* (144). Similar approach has demonstrated efficacy in a murine kidney model (24).

### Chronic Graft Dysfunction

Chronic lung allograft dysfunction (CLAD) remains a poorly understood process. Acute rejection, IRI leading to PGD, gastroesophageal reflux and recurrent infections are known risk factors for CLAD. In the current era, there is now emerging evidence that CLAD is related to aberrant airway repair, leading to airway remodeling, following airway epithelial injury. Th17 response is implicated in CLAD, and so is autoimmunity to various cryptic self-antigens.

Few investigators have utilized gene therapy to target CLAD, likely because few therapeutic targets are established for CLAD and a high fidelity animal model is lacking. IL-10 gene therapy has been delivered using multiple vectors—such as adenovirus, lentivirus, and sendai virus—in chronic rejection models and has been shown to attenuate bronchiolitis obliterans (BO) following lung transplantation (70, 145, 146). A combined *ex* and *in vivo* dual approach using lentiviral IL-10 was found to inhibit both acute and chronic rejection in a mouse model with decreased fibrosis and airway obliteration on histology (73). Jiang et al. successfully delivered hypoxia inducible factor 1α (HIF-1α) to the donor trachea by preserving the donor trachea in solution containing Ad vector, which attenuated airway fibrotic remodeling in a tracheal transplant model. HIF-1α transfection prevents loss of the epithelial lining and improves airway microvascular perfusion, while inducing expression of angiogenic factors such as PLGF and VEGF (147).

## Conclusion

Gene therapy has emerged as a potential therapeutic intervention in lung transplantation. Despite the interest in the field and overall positive experience in small and large animal models, the clinical translation of gene therapy in lung transplant remains limited. This is partly because most studies have been performed using Ad vectors, which have raised safety concerns in clinical trial and are now reserved for special indications. AAV is currently the leading platform for *in vivo* therapy; however, it has yet to be applied to lung transplantation. Transbronchial delivery of gene therapy is most widely used in experimental models and provides great transduction efficiency without off target effects. However, vascular delivery or novel modifications to the viral vectors may be necessary to target the vascular endothelium, which serves as the interphase between donor organ and recipient immune system. Several aspects of immune-mediated injuries in lung transplant can be targeted using gene therapy, including IRI (IL-10, HO-1, anti-apoptotic proteins), acute rejection (anti-inflammatory cytokines, co-inhibitory molecules, silence of immunogenic molecules) and CLAD. Ultimately, there is not a perfect one-for-all vector for gene therapy in lung transplant. There may, however, be an optimal vector for the delivery of a specific target gene of interest that will ensure sufficient transgene expression at the right time and in the right place.

For genes that target IRI, the protein product needs to be present at the time of reperfusion. Therefore, a vector such as an adenoviral vector with a fast onset is desired, and practically, organs will require a period of EVLP prior to implantation to facilitate protein synthesis. In contrast, if a late immune insult is targeted, it is preferred to use a vector that persists long-term and can evade the host immune system well. The recent popularization of EVLP has provided a unique platform for gene therapy delivery, but it remains to be seen whether this platform is necessary for all gene therapy.

Lastly, most pre-clinical gene therapy studies target one cytokine or immunomodulatory molecules. Practically, it is unlikely that modulation of a single pathway will solve complex processes such as IRI or CLAD. Co-administration of multiple vectors targeting multiple pathways may be necessary (148). Additionally, many cytokines are pleotropic with inhibition of some inflammatory pathways and activation of others. For example, IL-10 generally inhibits T_h_1 cytokines and NF-κB activity but promotes B cell proliferation and antibody production. TGF-β induces Tregs but promotes highly inflammatory T_H_17 cells. Therefore, constitutive expression of various cytokines may lead to undesired effects. Inflammation inducible vector has been reported in the literature and may potentially provide a solution (149).

## Author Contributions

QG, AB and MH conceived this review. QG, IA and ID preformed literature review and drafted the manuscript. IA, SK, NA, AA, RK performed critical review and revision of the manuscript. MH, AA and AB performed critical review and revision of the manuscript and oversaw the drafting process. All authors contributed to the article and approved the submitted version.

## Conflict of Interest

MH consults for Bridge to Life, Paragonix, Medtronic, and Intuitive Surgical and has research funding from Cystic Fibrosis Foundation, Biomedinnovations, and Transmedics. AA is co-founder and director at StrideBio and TorqueBio. He is also an advisor to Kriya Therapeutics, Atsena Therapeutics, Ring Therapeutics and Mammoth Bio.

The remaining authors declare that the research was conducted in the absence of any commercial or financial relationships that could be construed as a potential conflict of interest.

## Publisher’s Note

All claims expressed in this article are solely those of the authors and do not necessarily represent those of their affiliated organizations, or those of the publisher, the editors and the reviewers. Any product that may be evaluated in this article, or claim that may be made by its manufacturer, is not guaranteed or endorsed by the publisher.
